# Optimization of Donor Lymphocyte Infusion for AML Relapse After Allo-HCT in the Era of New Drugs and Cell Engineering

**DOI:** 10.3389/fonc.2021.790299

**Published:** 2022-01-27

**Authors:** Yishan Ye, Luxin Yang, Xiaolin Yuan, He Huang, Yi Luo

**Affiliations:** ^1^ First Affiliated Hospital, School of Medicine, Zhejiang University, Hangzhou, China; ^2^ Institute of Hematology, Zhejiang University, Hangzhou, China

**Keywords:** donor lymphocyte infusion (DLI), AML—acute myeloid leukemia, allogeneic hematopoietic cell transplantation, new drug, cell engineering

## Abstract

Donor lymphocyte infusion (DLI) is a key strategy for the treatment of AML relapse after allogeneic hematopoietic cell transplantation (allo-HCT) and has been used for either prophylactic, pre-emptive, or therapeutic purposes. However, the prognosis of these patients remains dismal even after DLI infusion (2-year overall survival, ~25%), and the efficacy is achieved at the cost of toxicities such as graft-versus-host (GVH) disease. Attempts to optimize DLI efficacy and safety, such as dose/timing modification and the use of cytoreduction, before DLI have been performed previously. Recently, a great number of novel targeted and immunomodulatory agents have emerged. Some of them, such as hypomethylating agents, FLT3 and Bcl-2 inhibitors, have been used in combination with DLI, aiming to enhance the graft-versus-leukemia effect. Moreover, manipulation of the DLI graft through cell selection (*e*.*g*., donor NK cells) or cell engineering (donor CAR-T cells) has shown potentially superior anti-tumor effects but less GVH effect than conventional DLI in clinical trials. This review summarizes the recent advances on the use of DLI for the prophylaxis/treatment of AML relapse and discusses future strategies which may further improve the treatment efficacy.

## Introduction

Allogeneic hematopoietic cell transplantation (allo-HCT) remains the therapy with the highest chance of long-term remission for acute myeloid leukemia (AML), especially for those in first complete remission (CR1) who belong to the European LeukemiaNet (ELN) intermediate or high-risk prognostic groups, those who remain measurable residual disease (MRD)-positive after induction therapy as well as those beyond CR1 (1). Disease relapse has been the main cause of failure for allo-HCT, with a dismal 2-year overall survival (OS) of less than 20% even in the most recent studies (1–4). Although both donor lymphocyte infusion (DLI) (5, 6) and a second allo-HCT (7, 8) have shown definite efficacy in patients deemed for intensive treatments, DLI seems to confer a similar outcome but with a lower incidence of non-relapse mortality (4). However, the survival benefits associated with DLI therapy remains unsatisfactory. In a study recently published, Kharfan-Dabaja et al. observed a 2-year OS of only 25% for patients with AML hematological relapse after allo-HCT who received therapeutic DLI (4). Therefore, there is still a lot of room for progress for DLI therapy in AML relapse after allo-HCT.

DLI is a kind of immunotherapy which can induce durable remission by enhancing the graft-versus-leukemia (GVL) effect (9). It was introduced for the treatment of leukemia relapse in the early 1990s (10) and was shown to be more effective in chronic myeloid leukemia (CML) (11, 12) than in acute leukemias (5, 13). With the application of tyrosine kinase inhibitors, DLI usage for CML relapse treatment has decreased. DLI could be used in either HLA-matched or mismatched allo-HCTs from different donors, including matched related, unrelated, or haploidentical related donors. With the introduction of anti-thymocyte globulin (ATG)-based and post-transplant cyclophosphamide (PT-Cy)-based haplo-HCT systems, the proportion of haploidentical HCT (haplo-HCT) within allo-HCT has increased rapidly (14, 15). Meanwhile, haplo-DLI has emerged as a promising strategy for leukemia relapse post-haplo-HCT since they may provide a stronger GVL effect due to a greater HLA disparity than those from HLA-matched donors (16, 17) and the fact that the availability of haploidentical related donors is superior to unrelated donors (9).

DLI has been used to either treat or prevent AML relapse. The therapeutic use of DLI is limited to T cell-replete allo-HCT protocols. Regarding the claim that DLI alone may not be sufficient to control overt relapse and that high tumor burden prior to DLI is associated with poor therapeutic response (4, 5, 18–20), cytoreduction with chemotherapy prior to therapeutic DLI (chemo+DLI) is frequently used, which may improve the efficacy and exert potential immunomodulatory effects (21, 22). Consistently, chemotherapy plus DLI has been found to be superior to DLI (23) or chemotherapy alone (13) in inducing disease remission after post-HCT AML hematological relapse. Pre-emptive and prophylactic DLIs have also been intensively investigated, which could effectively prevent hematological relapse with lower toxicity compared with therapeutic DLI. Pre-emptive DLI guided by MRD has shown definite efficacy in eliminating residual disease and promote donor chimerism (24, 25). Prophylactic DLI has been used to enhance immune reconstitution and prevent infection/relapse in T cell-depleted allo-HCT (26, 27) and in both ATG-based (28) and PT-Cy-based T cell-replete allo-HCT (29).

**Figure 1:**
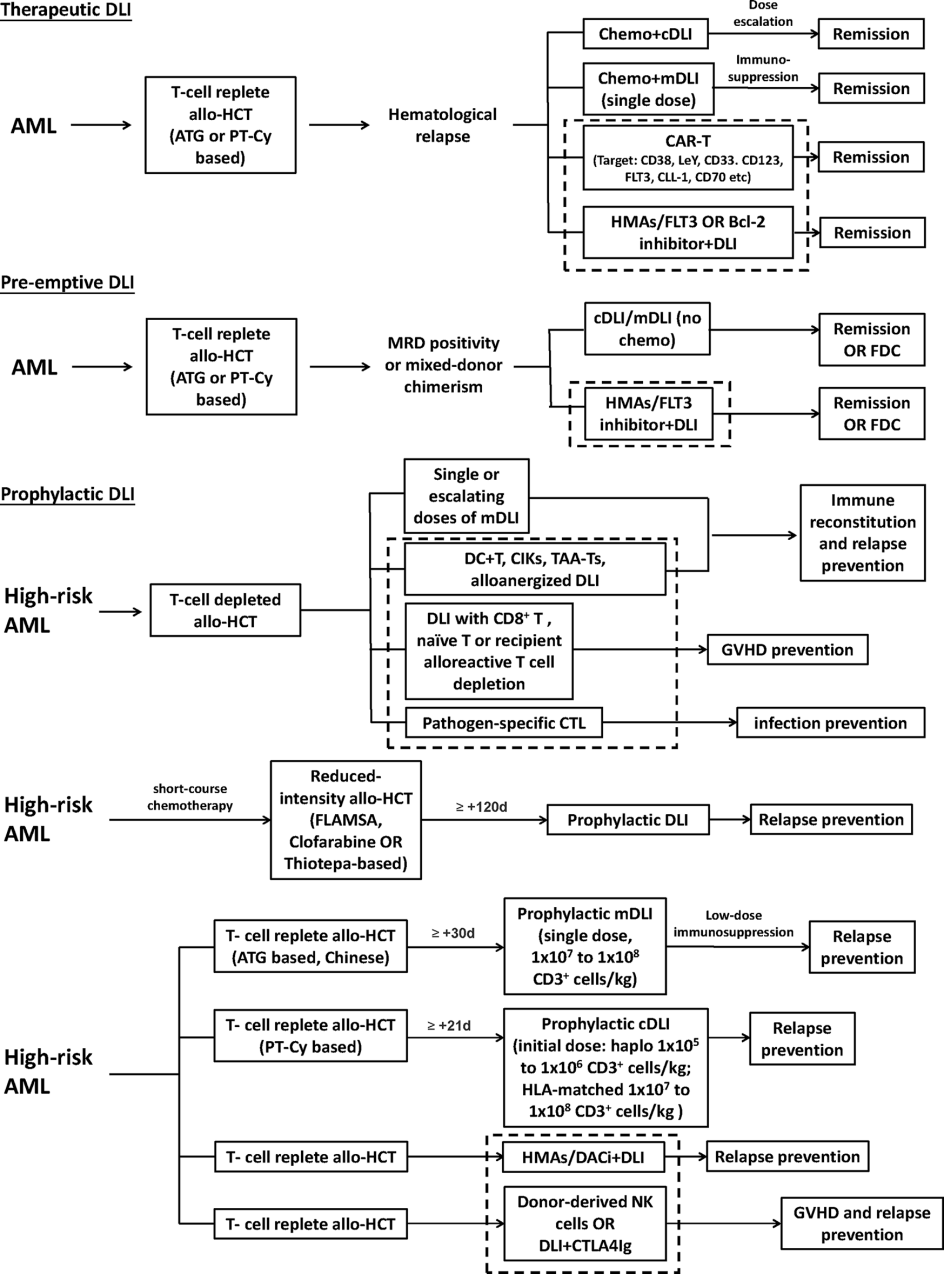
Recommended strategies to optimize the donor lymphocyte infusion (DLI), including DLI regimen and protocol improvement, additional use of novel drugs, and cell engineering. The area within the dotted bordered square indicate strategies, including novel drugs and cell engineering. High-risk AML represents those defined to have a higher risk of relapse in different studies. cDLI, DLI prepared by leukapheresis of unstimulated peripheral blood; mDLI, DLI derived from peripheral blood stem cells mobilized by granulocyte colony-stimulating factor; HMAs, hypomethylating agents; FDC, full donor chimerism; CIK, cytokine-induced killer cells; TAA-T, tumor-antigen-specific T cells; FLAMSA, condition regimen including fludarabine, cytarabine, and amsacrine.

In the recent decade, cellular therapy and novel drugs have come into focus for the treatment of hematological malignancies with exciting results. Efforts have been made to “transplant” these modalities to AML relapse after allo-HCT, with the aim to maximize the therapeutic effects while minimizing the toxicity. The recommended strategies to optimize the DLI, including DLI regimen and protocol improvement, additional use of novel drugs, and cell engineering, are schematically shown in **Figure 1**.

## Comparison of cDLI and mDLI

Two major types of DLIs are commonly used: the “conventional DLI” (cDLI in the following chapters) prepared by leukapheresis of unstimulated peripheral blood, and “modified DLI” (mDLI in the following chapters) using granulocyte colony-stimulating factor-mobilized peripheral blood stem cells (PBSCs). Both cDLI (5, 30) and mDLI (6, 31) have shown determined efficacy in the prophylaxis or treatment of AML relapse after allo-HCT. While cDLI is often used in Western countries using the PT-Cy-based allo-HCT protocol, mDLI, developed by Peking University, is regularly used by Chinese groups using the G-CSF/ATG-based protocol. Meanwhile, the cell dose is usually higher for mDLI (1 × 10^7^ to 1 × 10^8^ CD3^+^ cells/kg in both haplo and HLA-matched settings) (13, 32–34) than cDLI (haplo: 1 × 10^5^ to 1 × 10^6^ CD3^+^ cells/kg; HLA-matched: 1 × 10^7^ to 1 × 10^8^ CD3^+^ cells/kg) (35–37). Furthermore, mDLI is often followed by short-term immunosuppression with cyclosporine A (CsA) or methotrexate (38), while cDLI is not used in combination with GVHD prophylaxis (9). Comparative studies between cDLI and mDLI in the PT-Cy- or ATG-based allo-HCT protocols are required.

## Therapeutic DLI

Around 1995, inspired by a series of success for therapeutic DLI in CML (39), both cDLI (40) and mDLI (39) were evaluated in relapsed AML after allo-HCT. Collins et al. reported 39 AML patients receiving cDLI without chemotherapy after allo-HCT relapse; the CR rate was only 15.4% (40). With the addition of chemotherapy in prior, the CR rate and survival have increased. Schmid et al. summarized a big cohort of 399 AML patients with hematological relapse after allo-HCT: 75% of patients received chemo+cDLI, and the CR rate for the whole cohort was 34%, bringing an overall aGVHD incidence of 43%. Meanwhile, the 2-year OS was 21% for patients receiving cDLI and only 9% for those not receiving cDLI (5). Chemo+mDLI followed by short-term immunosuppression was developed by the group in Peking University and was majorly used after haplo-HCT (6). In an early report of 20 patients with relapsed hematological malignancies (AML, *n* = 7) after haplo-HCT, 9 received chemo+mDLI. Moreover, 75% (*n* = 15) of patients achieved complete remission, and the 2-year leukemia-free survival (LFS) was 40% for the whole cohort (6). Yan et al. evaluated the efficacy of chemo+mDLI in 82 patients (AML, *n* = 45) who relapsed after haplo-HCT. The CR rate was significantly higher in the chemo+mDLI cohort than in the chemotherapy cohort (64.0 vs. 12.5%) (13). The incidence of grade II–IV aGVHD was 62.7%. In this study, chronic GVHD (cGVHD) and durable MRD negativity after chemo+DLI were found to be associated with a lower relapse rate. Following this observation, a cGVHD- and MRD-guided chemo+DLI consolidation strategy was developed and tested in a prospective trial of 47 patients (AML, *n* = 25) (32). The 1-year relapse rate (22 vs. 56%) and LFS (71 vs. 35%) were significantly better in the consolidation cohort than in the control. More recently, therapeutic DLI has been investigated in the PT-Cy based haplo-HCT protocol. In a retrospective cohort of 40 patients (AML, *n* = 16) receiving cDLI-based therapy after relapse from PT-Cy-based haplo-HCT, 30% of patients achieved a CR, with 25% of patients developing aGVHD. Meanwhile, higher rates of CR were achieved among those treated with chemo+cDLI than cDLI alone (41). A study conducted by Ghiso et al. used either cDLI alone or chemo+cDLI for molecular or hematological relapsed leukemia patients after PT-Cy-based haplo-HCT. A response rate of 33% and grades II and III aGVHD incidence of 17% were reported (24). Goldsmith et al. summarized 21 patients with disease relapse (90%) or loss of chimerism (10%) after PT-Cy-based haplo-HCT, in which 76% of patients received chemo+cDLI. Seven patients (33%) finally achieved CR or full donor chimerism, and the grades I–III aGVHD incidence was 23.8% (42). The experience on therapeutic DLI is limited to ATG- or PTCy-based T cell-replete allo-HCT protocols.

## Pre-emptive DLI

MRD positivity prior to (43) or after (44) allo-HCT is positively associated with relapse incidence and negatively correlated with disease-free survival for AML patients. Furthermore, early MRD after allo-HCT (within +30 day) predicts the highest risk of relapse (44). Meanwhile, loss of donor chimerism (mixed chimerism, MC) predicts disease relapse and poorer relapse-free survival after allo-HCT in AML (45, 46). Pre-emptive DLI has been used to eradicate MRD and promote donor chimerism after allo-HCT in AML.

Pre-emptive DLI has brought about definite benefits in response rate and survival as compared to therapeutic DLI (24, 25, 47). Ghiso et al. compared pre-emptive cDLI for molecular relapse and therapeutic cDLI for hematological relapse after PT-Cy-based haplo-HCT. A higher response rate in the pre-emptive cohort (45 vs. 33%) was observed (24). Recently, Rettig et al. reported a significantly better survival for post-allo-HCT AML patients receiving pre-emptive than therapeutic mDLI (2-year OS: 64 vs. 26%) (25).

MRD is the most frequently used indicator for pre-emptive DLI, which is detected majorly by either flow cytometry (47), quantitative PCR (qPCR) (48), or, more recently, genomic sequencing (49). Yan et al. compared pre-emptive mDLI and IL-2 therapy on 105 standard-risk acute leukemia patients (AML, *n* = 61) with persistent MRD after allo-HCT. The 3-year cumulative incidence (CI) of relapse was significantly lower (27.8 vs. 64.4%), and the disease-free survival (55.6 vs. 24.1%) was significantly better in the pre-emptive mDLI cohort (18). The grade II–IV aGVHD and extensive cGVHD incidences after pre-emptive mDLI were 27.9 and 34.2%, respectively. Since patients with late-onset MRD had a lower risk of relapse than patients with early-onset MRD after pre-emptive DLI (50), we further compared the relapse rate and survival between pre-emptive DLI and IL-2 in patients with late-onset MRD (MRD positivity at >100 days after allo-HCT), and similar outcomes for the two strategies were observed (51). Quantitative assessment of WT1 as MRD has been used as a marker for pre-emptive DLI in AML patients after allo-HCT (52), and WT1 copies/10^4^ Abelson cells in marrow cells has been suggested as the cutoff value (48). In order to better prevent disease recurrence, chemo+mDLI as pre-emptive therapy has been evaluated in ATG-based T cell-replete allo-HCT (53). A comparative study between chemo+pre-emptive mDLI and pre-emptive mDLI alone in acute leukemia/MDS patients showed that both strategies led to similar percentages of patients (chemo+DLI: 78.3 vs. DLI: 75%) to turn MRD-negative, thus advocating the use of pre-emptive mDLI alone for MRD^+^ patients for its lower toxicity. Several studies have also shown that pre-emptive DLI effectively converts MC to full donor chimerism (FDC), while its capacity in preventing disease relapse or prolonging survival remains to be established for lack of comparative studies (54). Caldemeyer et al. reported pre-emptive cDLI use in 29 patients with hematological malignancies who were identified MC without detectable disease after allo-HCT, and 93% of these patients converted to FDC with a cumulative grade II–IV aGVHD and/or extensive cGVHD incidence of 31% (55). Feliu et al. retrospectively analyzed 119 patients (AML, *n* = 48) receiving pre-emptive cDLI for falling CD3 chimerism, and 60% of patients achieved FDC after treatment (56). Of note is the fact that, in relapsed patients with exclusively recipient chimerism, DLI may not be effective and may lead to severe aplasia (57).

## Prophylactic DLI

### Prophylactic DLI in T Cell-Depleted Allo-HCT

Allo-BMT or HCT with *ex vivo* T cell depletion has been carried out extensively from the 1980s in both HLA-identical (58) or haploidentical settings (59). *Ex vivo* T cell depletion minimizes GVHD but is associated with slow immune recovery and a higher risk of relapse and infections (60). Prophylactic DLI has been used to enhance immune reconstitution so as to reduce the infection and relapse rates (26, 27). Kothari et al. studied, in a prospective trial, 75 patients (AML, *n* = 37) receiving T cell-depleted-matched related allo-HCT, in which 26 patients received at least one cDLI (median DLI number ≥3). The 2-year PFS was better in the DLI cohort than the entire population (57 vs. 41%), and the 1-year grade II–IV aGVHD rate was 28% for the DLI cohort (61). Gilman et al. studied prophylactic DLI after T cell-depleted haploidentical HCT in a phase I/II trial comprising 35 patients (AML, *n* = 10) (27). These patients received between day +30 and day +42 a single dose of up to 5◊10^4^ CD^3^/kg prophylactic mDLI, followed by methotrexate; a 2-year OS of 69% was achieved, with a CI of 11% for grade II–IV aGVHD and 14% for cGVHD (27). Several techniques have been applied to manipulate prophylactic DLI to enhance its anti-leukemic and anti-infectious capacity while sparing the GVH effects. Following the rational that CD8^+^ T cell depletion reduces the risk of GVHD induced by DLI (62), Dodero et al. reported the results of a phase I/II trial comprising 23 patients with hematological malignancies who received escalating doses of prophylactic CD8^+^ T-depleted DLI (median DLI number = 2) after T cell-depleted reduced-intensity allo-HCT. A grade II–IV aGVHD incidence of 26% was observed (63). Regulatory T cells (Tregs) could prevent GVHD occurrence by facilitating the immune self-tolerance and homeostasis (64), which have been co-infused to suppress the GVH while maintaining the GVL effects (65). Martelli et al. reported 43 acute leukemia patients (AML, *n* = 33) receiving T cell-depleted haplo-HCT. These patients received freshly isolated donor Tregs on day -4, followed by a megadose of purified CD34^+^ cells and Tcons on day 0. Only 15% of the patients developed grade II–IV aGVHD, with a CI of 5% after 46 months of follow-up (66). Recently, the same group has updated the data using this transplant protocol in 50 aged AML patients (age >50) receiving haplo-HCT. Fifteen (30%) patients developed grade II–IV aGVHD, and only 2 patients (4%) relapsed. The OS after a median follow-up of 29 months was 77% (67). Infusion of pathogen-specific T cells may decrease the risk of specific infections after transplantation. Perruccio et al. generated and infused donor alloantigen-deleted Aspergillus and CMV-specific donor T cells after T cell-depleted haplo-HCT. These two adoptive immunotherapy strategies successfully cleared invasive aspergillosis and prevented CMV reactivation, respectively (68). Recently, third-party pathogen-specific cytotoxic T cells have been developed for off-the-shelf use. Olsen et al. reported a trial using third-party BK virus-specific cytotoxic T cell infusions into 59 patients developing BK virus-associated hemorrhagic cystitis after allo-HCT, and a day 14 overall response rate of 67.7% was observed (69). Moreover, several techniques are being investigated for optimal T cell allodepletion with preservation of pathogen-specific responses and Tregs (70). Despite these encouraging results, cell selection remains a time- and labor-consuming technique with high expenses, which has limited its extensive use.

### Prophylactic DLI in T Cell-Replete Allo-HCT

Schmid et al. reported an early trial testing of prophylactic cDLI in 75 patients with high-risk AML/MDS defined by progressive/refractory (R/R) disease, second remission after early relapse, or first remission with poor cytogenetics/delayed response to induction chemotherapy. These patients received reduced-intensity T cell-replete HLA-matched allo-HCT. Escalating doses of prophylactic cDLI without immunosuppression were infused from day +120 for patients without immunosuppression or GVHD (matched related donor, escalating from 1◊10^5^ to 2◊10^6^ CD3^+^ cells/kg; matched unrelated donor, escalating from 2◊10^5^ to 5◊10^6^ CD3^+^ cells/kg, median DLI number = 2). A 2-year LFS of 40% was achieved (71). Later, Jedlickova et al. compared the long-term results of patients receiving prophylactic cDLI within the prospective trial introduced above with a well-matched control group without DLI. The overall survival at 7 years after transplant was 67%, compared with 31% in the control group (*P* < 0.001). This study has demonstrated the long-term survival benefit achieved by prophylactic cDLI in AML/MDS (37). Indeed the prophylactic cDLI usage described above is part of a sequential approach combining (1) a short course of chemotherapy followed by a reduced-intensity conditioning regimen (2), reduced-intensity allo-HCT (3), new drugs to prevent relapse, and (4) prophylactic DLI starting at day +120. This sequential regimen aims to reduce the toxicity of allo-HCT and optimize the anti-leukemia effects of DLI. In the two studies described above, the FLAMSA regimen (fludarabine, cytarabine, and amsacrine) was applied. Similarly, other allo-HCT protocols have also been reported as part of this sequential regimen (72). Mohty et al. conducted a phase II study testing a sequential regimen of clofarabine, cytosine arabinoside, and reduced-intensity allo-HCT, followed by delayed prophylactic cDLI, in refractory AML patients (73). The 1-year OS and CI of non-relapse mortality (NRM) were 54 and 8%, respectively. Shelikova et al. reported 22 children with R/R AML who received cytoreduction with fludarabine and cytarabine and subsequent myeloablative conditioning with treosulfan and thiotepa. The following prophylactic mDLI or cDLI comprised of a CD45RA-depleted fraction with or without a hypomethylating agent. The CR rate was 95%, with a 2-year EFS of 49% and TRM of 9% (74).

The Chinese groups evaluated extensively prophylactic mDLI in the ATG-based T cell-replete protocol, which usually included a single mDLI dose (the same for haplo and HLA-matched settings) followed by short-term immunosuppression. Wang et al. retrospectively compared patients receiving prophylactic mDLI and those without in T cell-replete haplo-HCT (median 0.6◊10^8^ CD3^+^ cells/kg for the entire cohort). The 2-year CI of relapse was lower (36 vs. 55%) and the 3-year OS was better (31 vs. 11%) in the prophylactic DLI group than in the control (28). Later, the same group developed a strategy of prophylactic mDLI followed by MRD- and GVHD-guided multiple DLIs for R/R acute leukemia. In a prospective study comprising 100 patients (AML, *n* = 59), this protocol achieved a 3-year CI of relapse, LFS, and OS of 32, 50, and 51%, respectively (33). We have recently reported a matched-pair study in ATG-based haplo-HCT between patients (*n* = 34; AML, *n* = 28) receiving a single-dose prophylactic mDLI (median: 3.8◊10^7^ CD3^+^ cells/kg), followed by low-dose CsA, and the same number of patients without. The 5-year LFS was superior (65 vs. 34%), and the CI of relapse (15 vs. 49%) was lower for the mDLI cohort than the control. Meanwhile, the 100-day CI of grade II–IV aGVHD for the mDLI cohort was 26.7% (34). Gao et al. compared haploidentical and matched-sibling mDLI, followed by low-dose CsA (median: 2.3◊10^7^ CD3^+^ cells/kg for the entire cohort), in high-risk AML defined by progressive disease at transplant, CR1 achievement with ≥3 cycles of chemotherapy, or those carrying TP53, DNMT3a, TET2, or FLT3-ITD gene mutations. They observed a higher 100-day grade II–IV aGVHD CI (60 vs. 31%) and worse 1-year NRM (28 vs. 0%) in the haplo than the matched-sibling cohort, indicating a potentially higher toxicity for haplo than matched-sibling DLI (75).

Prophylactic DLI has also been evaluated in PT-Cy-based T cell-replete allo-HCT. Jaiswal et al. reported a small cohort of 21 AML patients not in remission who underwent PT-Cy-based haplo-HCT and received three consecutive prophylactic mDLIs, with each dose being 1 × 10^6^ CD3^+^ cells/kg, on days +21, +35, and +60 with concurrent CsA use (71% of the studied patients received all 3 doses), which decreased the progression rate from 66 to 21% (29). Cauchois et al. analyzed 36 patients with high-risk hematological malignancies (AML, *n* = 21), defined as with a high disease risk index or not in CR before transplant who received escalating doses of prophylactic cDLI (from 1 × 10^5^/kg to 2.5 × 10^6^/kg, median DLI number = 1) after PT-Cy-based haplo-HCT. The CI of relapse at 1 year after prophylactic DLI was 16%, and the PFS was 76%, respectively (76).

## Feasibility and Side Effects of DLI

Although DLIs have shown definite efficacy in preventing or controlling disease relapse after allo-HCT, feasibility requires to be examined before each DLI infusion even if it is planned and the donor is willing to donate. Prophylactic or pre-emptive DLI may not be administrated because of early relapse, a history of severe GVHD, active GVHD with continuous need of immunosuppression, severe infections, significant cytopenia, early death or choice of the patients (37). In the trial conducted by Jedlickova et al. testing prophylactic DLI for high-risk AML after allo-HCT, only ~30% of patients received prophylactic DLI as planned in the protocol (37), while in another phase II trial on prophylactic azacytidine + DLI for high-risk AML/MDS after allo-HCT, 56.7% of patients received at least one DLI (36). In the retrospective study conducted by Guillaume et al. evaluating the efficacy of prophylactic/pre-emptive azacytidine + DLI on high-risk AML/MDS after allo-HCT, 79% of patients received at least one DLI (77). For therapeutic DLI, inadequate organ function, active severe acute/chronic GVHD, and severe infections are regarded as contraindications (24). Another crucial barrier for successful DLI usage is HLA loss, which represents an important immune-escape mechanism that allows the leukemia to relapse after allo-HCT (78). Genomic loss of mismatched HLA occurs through a mechanism of copy-neutral loss of heterozygosity, eliminating the incompatible HLA alleles without decreasing the overall level of expression of HLA class I molecules (79). HLA loss accounts for around one-third of relapses after haplo-HCT (78, 80) and also a minority of relapses from matched or mismatched unrelated allo-HCTs (81). Since patients with HLA loss relapse were unlikely to benefit from DLI of the original donor, the 2019 EBMT consensus of DLI after haplo-HCT recommended a second allo-HCT from a related donor with a different mismatched haplotype or a mismatched unrelated donor (9).

GVHD is the main complication of DLI. The morbidity and severity of DLI-related GVHD was closely related to the type of DLI (cDLI or mDLI), the time interval between transplantation and DLI, the transplant protocol, the dose of CD3^+^ cells, the donor type, and the immunosuppressant. Since G-CSF mobilization alters the cellular composition and cytokine profile of the DLI graft, it was suggested that the mDLI might reduce the GVHD incidence (38), but this hypothesis is not yet confirmed in a head-to-head study. The time interval between transplantation and DLI greatly affects the morbidity and severity of DLI-associated GVHD (34). It is accepted that the shorter the interval, the higher the risk that GVHD will occur. In our study, we initially tested prophylactic mDLI in 5 patients at a median of 71 days post-HCT; 2 patients developed grade IV aGVHD, and 1 had grade I aGVHD, indicating a high incidence of aGVHD for early prophylactic mDLI infusion (31). Su et al. compared the safety of efficacy of prophylactic mDLI on day +60 and day +90 after T cell-replete allo-HCT and observed that prophylactic mDLI on day +90 was associated with a lower extensive cGVHD incidence (10 vs. 28%) and better GVHD-free, relapse-free survival (GRFS) (55 vs. 41%) (82). Meanwhile, pre-emptive/prophylactic DLI infused very early after allo-HCT (within 40 days) may be impaired by the remaining ATG in the human body (83).

In different transplant protocols, the timing of DLI and the related GVHD incidence vary greatly. In an earlier study conducted by Liga et al., 15 patients (AML, *n* = 8) received HLA-identical allo-HCT with an alemtuzumab-containing conditioning regimen and prophylactic mDLI with dose escalation (dose escalation for unrelated donor: 0.5 to 1 × 10^6^ CD3^+^ cells/kg, for sibling: 0.75 to 1.5 × 10^6^ CD3^+^ cells/kg, median DLI number = 3) in the absence of concurrent immunosuppression. The first infusion was given at a median of 162 days post-transplantation, and the DLI-associated GVHD incidence was 47% (84). In the PT-Cy-based haplo-HCT, an earlier study conducted by Legrand et al. included 22 AML patients receiving escalating doses of prophylactic cDLI (median DLI number = 1) after HLA-matched or haploidentical HCT, in which 4 (18%) received PT-Cy as GVHD prophylaxis. The first prophylactic DLI (the dose ranged from 0.1 to 10 × 10^6^ CD3^+^/kg) was performed in a median time of 130 days post-transplant, and the cumulative incidence of DLI-associated GVHD was 37% (35). Later, Cauchois et al. reported 36 patients receiving prophylactic cDLI with dose escalation after PT-Cy-based haplo-HCT (escalating from 1 × 10^5^/kg to 2.5 × 10^6^ CD3^+^ cells/kg, median DLI number = 1) at a median time of 109 days between transplant and the first DLI; the cumulative 1-year incidence of cDLI-induced GVHD was 33% (76). Notably, in the study conducted by Jaiswal et al., the first prophylactic mDLI was given as early as +21 day after PT-Cy-based haplo-HCT with concurrent immunosuppression, and the cumulative incidence of aGVHD was only 31% (29). This study indicates that concurrent GVHD prophylaxis might control the GVHD caused by very early DLI infusions.

The CD3^+^ T cell dose is another important factor affecting DLI-associated GVHD. An early comparative study between escalating cDLI and a single infusion in relapsed CML after allo-HCT showed that the escalating regimen was associated with a lower incidence of GVHD but with similar outcomes in disease control (85). Bar et al. retrospectively analyzed the effects of an initial CD3^+^ T cell dose within therapeutic cDLI on the GVHD incidence after allo-HCT in 225 patients with hematological malignancies (AML, *n* = 71). Compared to lower doses, an initial CD3^+^ T cell dose above 1 × 10^8^/kg in cDLI was found to be associated with a higher 1-year CI of GVHD (55%) but did not decrease the relapse incidence or improve the survival (86). As for donor type, haplo-DLI may bring about a higher incidence of GVHD than HLA-matched DLI due to higher alloreactivity. Gao et al. observed a higher incidence of grade II–IV DLI-associated aGVHD in haploidentical than matched sibling prophylactic mDLI (60 vs. 31%) after allo-HCT in high-risk AML (2◊10^7^ CD3^+^ cells/kg for both groups) (75). Yu et al. analyzed refractory acute leukemia patients receiving allo-HCT followed by prophylactic mDLI (3◊10^7^ CD3^+^ cells/kg for both groups). A total of 119 patients (AML, *n* = 54) received haploidentical and 132 patients (AML, *n* = 57) received matched-sibling donor allo-HCT. The haploidentical group was associated with a higher incidence of grade II–IV aGVHD (62 vs. 54% p=0.025) but with a similar 3-year incidence of cGVHD (87).

Concurrent immunosuppression has been routinely used with mDLI in the G-CSF/ATG-based allo-HCT protocol to reduce the incidence of DLI-associated GVHD. Immunosuppression with either CsA or methotrexate (MTX) is normally used right after a single dose of mDLI and maintained for 2–6 weeks according to the donor–recipient relationship, the aim of the DLI (prophylactic, pre-emptive, or therapeutic), or at the discretion of the physician. We reported the use of low-dose CsA (1 to 2 mg/kg/day) after DLI for 3 to 4 weeks after prophylactic mDLI (median of 3.8 × 10^7^ CD3^+^ cells/kg) in the G-CSF/ATG-based allo-HCT protocol, and the CI of grade II–IV DLI-associated aGVHD was 17.6% (34). Yan et al. reported GVHD prophylaxis for 2–4 weeks after HLA-identical related pre-emptive mDLI or 4–6 weeks after an HLA-identical unrelated or HLA-haploidentical mDLI. The grade II–IV DLI-associated aGVHD incidence was 28% (18). When comparing CsA and MTX, MTX might bring about a similar anti-GVHD effect while preserving a stronger GVL effect due to a higher maintenance of absolute lymphocyte count (88). In a retrospective study including 124 patients receiving either prophylactic, pre-emptive, or therapeutic DLI, Yan et al. observed that the duration of GVHD prophylaxis was the only independent factor affecting the development of grade III–IV mDLI-associated aGVHD. The cumulative incidences of grade III–IV acute GVHD in patients with prophylaxis more than 6, 4–6, 2–4, and <2 weeks were 9.3, 14.4, 31.6, and 49.5%, respectively (*p* = 0.018) (89). In PT-Cy-based myeloablative haplo-HCT, Jaiswal et al. reported CsA use during consecutive prophylactic mDLIs (each dose: 1 × 10^6^ CD3^+^ cells/kg) until day +60 and tapered over 4 weeks. The cumulative incidence of aGVHD was 31% (29). It is noteworthy that dose escalation strategy is frequently used with cDLI according to the CD3^+^ cell count for GVHD prevention, while adjuvant immunosuppression has not been reported. Meanwhile, no comparative study has supported differences on the incidences of DLI-associated aGVHD between mDLI plus immunosuppression and cDLI without immunosuppression (9). Finally, the possibility of impaired GVL effect after immunosuppression remains to be excluded.

Aplasia happens in between 18 and 40% of patients after DLI infusion (24, 38). Insufficient donor chimerism and conditions of DLI usage might be important reasons for the development of aplasia (57). Prophylactic (34, 37, 75, 90) and pre-emptive DLIs (56) seem to rarely induce DLI-associated cytopenia, while therapeutic DLIs are associated with a considerable risk of aplasia. Glass et al. reported both neutropenia and thrombocytopenia incidences of 36% among 11 patients (AML, *n* = 5) with hematological malignancy relapse after allo-HCT who received cytoreductive therapy+mDLI (39). Huang observed incidences of 20% neutropenia and 35% thrombocytopenia among 20 patients (AML, *n* = 7) with hematological malignancy relapse after allo-HCT and therapeutic DLI (6). The higher incidence of cytopenia during therapeutic DLI is probable due to cytoreductive therapy *in prior* and lower donor chimerism during infusions. Notably, DLI with adjuvant drugs has also been reported to induce considerable hematological toxicities. Grade III/IV neutropenia and thrombocytopenia occurred during 65 and 63% of treatment cycles in a prospective single-arm multicenter phase II trial, conducted by Schroeder et al., testing azacytidine plus therapeutic cDLI for AML/MDS relapse after allo-HCT (91). Similarly, venetoclax plus therapeutic mDLI led to anemia, neutropenia, and thrombocytopenia in 55, 73, and 64% of patients with relapsed AML after allo-HCT, respectively (92). However, it is important to clarify the reason for cytopenia since it could be due to other reasons than DLI itself, including primary disease, infection, GVHD, *etc.*

Infection is another non-negligible complication after DLI following DLI-associated GVHD or cytopenia. Viral and fungal infections are main causes of infection-related deaths (71). In T cell-depleted haplo-HCT, Gilman et al. reported that 11% of patients died of fatal viral/fungal infections after prophylactic cDLI (27). In the ATG-based T cell-replete protocol, an earlier study reported a high septicemia incidence of 49% after prophylactic cDLI and an infection-related death incidence of 25% at 1 year after transplant (71). With the advancement of anti-infection strategies, in a recent study conducted within the ATG-based T cell-replete allo-HCT protocol, Su reported an infection-related death rate of 10–15% after prophylactic mDLI (82). In the FLAMSA RIC protocol, Jedlickova reported an incidence of only 4% for infection-related death after prophylactic cDLI after 7 years of follow-up. For therapeutic DLI, Huang et al. previously observed that therapeutic mDLI led to an infection-related death incidence of 20% after T-cell replete ATG. More recently, Rettig et al. reported an infection-related death incidence of only 2% after therapeutic mDLI usage for AML relapse after ATG-based allo-HCT (25). In the PT-Cy-based T cell-replete protocol, Jaiswal documented 5 (24%) CMV infections and 1 (5%) fungal infection among 21 patients receiving PT-Cy-based haplo-HCT and prophylactic mDLI (29). Moreover, Ghiso et al. reported a cumulative infection incidence of 12% after therapeutic or pre-emptive cDLI after PT-Cy-based haplo-HCT (24). Collectively, regardless of the great difference among transplant platforms, advanced infection management strategies may have significantly reduced the incidences of infection-related death after therapeutic DLI.

## New Drugs and DLI

### Hypomethylating Agents

The clinical studies on the combined use of novel drugs and DLI are summarized in **Table 1**. Hypomethylating agents azacitidine and decitabine are approved drugs with demonstrated efficacy and prolong the survival of MDS and AML patients (102). Early preclinical studies showed that low-dose azacitidine upregulates epigenetically silenced tumor antigens and induces a cytotoxic T cell response (103), and it also mitigates GVHD *via* Treg induction (104). The hypomethylating agent (HMA)/DLI combination has been used for both salvage therapy and post-transplant maintenance in AML/MDS. In a multi-center retrospective analysis comprising 154 relapsed AML/MDS (AML, *n* = 124; MDS, *n* = 28; MPN, *n* = 2) patients receiving HMA/DLI combination, the overall response rate (ORR) was 33%, and the CR rate was 27% (93). In a bigger retrospective study derived from the EBMT database comprising 181 patients (AML, *n* = 116; MDS, *n* = 65) receiving HMA/DLI combination as salvage therapy after hematological relapse, the ORR rate was 29%, and the CR rate was 15% (94). Schroeder et al. reported 30 relapsed AML/MDS (AML, *n* = 28, MDS, *n* = 2) patients receiving up to 6 cycles of azacytidine, followed by DLI after every second cycle in a prospective trial. The overall response rate (ORR) was 30%, and the CR rate was 23% (91). Moreover, following the pre-clinical evidence that azacitidine mitigates GVHD, Ghobadi et al. conducted a phase I trial wherein azacitidine was applied on days 4, 6, 8, and 10 post-DLI in 8 patients with post-transplant AML relapse; 6 out of 8 (75%) patients responded with 2 (25%) cytogenetic complete remissions. Of note is the fact that no patient experienced grade III–IV aGVHD, indicating enough safety and GVHD prevention capacity for azacitidine use following DLI (95).

**Table 1:** Published studies on new drugs/DLI combined use for AML relapse after allo-HCT.

Study	No. of Patients (Perspective/Retrospective)	Diagnosis/Disease Status Before Therapy	Drug	Total Dose/Dose of Each Course	Indication for DLI	Median TotalCD3+/kg	Median Number of DLI	Disease Response	GVHD	Survival	Notes
**Hypomethylating agents/DLI combination**											
Schroeder et al. (91)	30 (prospective)	AML = 28 MDS = 2 Hematological relapse	Aza	Median course number = 3 Each course: 100 mg/m^2^ × 5 days	Therapeutic	5 × 10^6^	1	Aza and Aza+DLI: ORR = 30% CR = 23%	Aza and Aza+DLI: aGVHD = 37% cGVHD= 17%	OS after median of 817 days: 17%	DLI after every 2nd Aza cycle
Schroeder et al. (93)	154 (retrospective)	AML = 128 MDS = 28 MPN = 2 Hematological/molecular relapse	Aza	Median course number = 4; each course: 100 mg/m^2^ × 5 days or 75 mg/m^2^ × 7 days	Therapeutic	31.2 × 10^6^	2	Aza+DLI: ORR = 33% CR = 27%	Aza+DLI: aGVHD = 31% cGVHD = 31%	2-year OS: molecular relapse: 69%; hematological relapse: 19%	DLI after every 2nd Aza cycle
Craddock et al. (94)	181 (retrospective)	AML = 116 MDS = 65 Hematological relapse	Aza	1,050 mg/m^2^	Therapeutic	N.A.	N.A.	Aza and Aza+DLI: ORR = 29.3% CR = 15.3%	Aza and Aza+DLI: grade II–IV aGVHD = 7%	Aza alone or Aza+DLI: 2-year OS: 12.4%	
Ghobadi et al. (95)	8 (prospective)	AML = 8 Hematological relapse	Aza	Each infusion: 3 received 45 mg/m^2^; 5 received 75 mg/m^2^	Therapeutic	1 × 10^7^	1	Aza+DLI: ORR = 75% (6/8) CR = 37.5% CRi = 37.5%	Aza+DLI: aGVHD = 62.5%	Aza+DLI: median OS: 12.5 months; median DFS: 2.9 months	Aza on days 4, 6, 8, and 10 after DLI
Guillaume et al. (36)	30 (prospective)	AML = 20 MDS = 10 Hematological remission	Aza	Median course number = 5 Each course: 32 mg/m^2^× 5 days	Prophylactic	N.A.	3	Aza and Aza+DLI: 2-year CI of relapse = 27.6%	Aza and Aza+DLI: Grades I–III aGVHD = 31.5% 2-year cGVHD = 53%	Aza and Aza+DLI: 2-year OS: 65.5% 2-year DFS: 65.5%	
Guillaume et al. (77)	77 (retrospective)	AML = 54 MDS = 23 Hematological remission	Aza	Median course number = 9 Each course: 32 mg/m^2^ × 5 days	Pre-emptive or prophylactic	N.A.	1	Aza and Aza+DLI: 2-year CI of relapse = 22%	Aza and Aza+DLI: grade II–IV aGVHD: 27.4% cGVHD = 45%	Aza and Aza+DLI: 2-year OS: 70.8% 2-year PFS: 68.3%	
Schroeder et al. (96)	36 (retrospective)	AML = 29 MDS = 7 Hematological/molecular relapse	Dac	Median course number = 2 Each course: 20 mg/m^2^ × 5 days or 10 days	Therapeutic	6.5 × 10^6^	2	Dac and Dac+DLI: ORR = 25% CR = 17%	Dac and Dac+DLI: aGVHD: 19% cGVHD = 5%	Aza and Aza+DLI: 2-year OS: 11%	
Sommer et al. (97)	26 (retrospective)	AML = 18 MDS = 6 MPN = 2 Hematological relapse	Dac	Median course number = 2 Each course: 20 mg/m^2^ × 5 days or 10 days	Therapeutic	N.A.	2	Dac and Dac+DLI: ORR = 19% CR/Cri = 15%	Dac+DLI: aGVHD: 17% cGVHD = 6%	Median OS: 4.7 months	
Zhang et al. (90)	28 (prospective)	AML = 23 MDS = 2 ALL = 3	Dac	Median course number = 2 Each course: 10 mg/m^2^×5d	Prophylactic	2 × 10^7^	1	DLI and Dac+DLI: 3-year CI of relapse = 26.1%	DLI and Dac+DLI: 100-day aGVHD: 25.8% 3-year cGVHD: 21.6%	DLI and Dac+DLI: 3-year OS: 48.9% 3-year RFS: 48.2%	mDLI
**FLT3 inhibitors**											
Xuan et al. (98)	41 (retrospective)	AML with FLT3-ITD Hematological relapse	Sorafenib	400 mg twice daily, adjusted on suspected toxicity	Therapeutic	3.2 × 10^7^	1	Sorafenib+chemo+DLI: ORR: 87.8% CR: 80.7%	Sorafenib+chemo+DLI: 1-year aGVHD: 39.5% 1-year cGVHD: 32.8%	Sorafenib+chemo+DLI: 1-year OS: 53.2% 3-year RFS: 50.8%	mDLI
Bruzzese et al. (99)	4 (retrospective)	AML with FLT3-ITD MRD-positive	Sorafenib	200 mg twice daily	Pre-emptive	22.6 × 10^6^	3	Sorafenib+DLI: MRD negativity: 75% Relapse incidence: 0%	Sorafenib+DLI: aGVHD: 0% cGVHD: 25%	Sorafenib+DLI: After 38.7 months OS: 100% PFS: 100%	
**Bcl-2 inhibitors**											
Amit et al. (92)	22 (retrospective)	AML = 22 Hematological/molecular relapse	Venetoclax	Median course number = 2 Each course: 400 mg daily × 28 days	Therapeutic	N.A.	1	Venetoclax+DLI: ORR: 50% CR/CRi: 23%	Venetoclax+DLI: aGVHD: 18% cGVHD: 27%	Venetoclax+DLI: Median OS: 6.1 months	
**Deacetylase inhibitors**											
Bug et al. (100)	18 (prospective)	AML (unknown) MDS (unknown)	Pnb	Schedule A: 10 mg TIW weekly Schedule B: 20 mg TIW every other week	Prophylactic	Schedule A: 0.2 × 10^6^ Schedule B: 0.9 × 10^6^	2	N.A.	N.A.	N.A.	
Kalin et al. (101)	110 (prospective)	AML = 110	Pnb+Dac		Prophylactic	N.A.	2	Pnb+Dac and Pnb+Dax+DLI: 2-year CI of relapse = 35%	Pnb+Dac and Pnb+Dac+DLI 6-month grade II–IV aGVHD: 23% 1-year moderate–severe cGVHD: 22%	Pnb+Dac and Pnb+Dac+DLI: 2-year OS: 50% 2-year PFS: 49%	

DLI, donor lymphocyte infusion; AML, acute myeloid leukemia; MDS, myelodysplastic syndromes; Aza, azacytidine; ORR, overall response rate; CR, complete remission; aGVHD: acute graft-versus-host disease; cGVHD: chronic graft-versus-host disease; OS, overall survival; MPN, myeloproliferative neoplasms; Cri, CR with incomplete count recovery; DFS, disease-free survival; CI, cumulative incidence; PFS, progression-free survival; Dac, decitabine; RFS, relapse-free survival; mDLI, mobilized DLI from G-CSF mobilized PBSC; MRD, minimal residual disease; Pnb, panobinostat; N.A., Not available.

Guillaume et al. conducted a phase II trial evaluating prophylactic azacytidine followed by DLI in 30 high-risk AML/MDS patients. High-risk AML was defined as CR1 with unfavorable cytogenetics, requiring ≥2 cycles of treatment for remission, remission greater than CR2, or progressive disease. High-risk MDS was defined as with an intermediate-2 or higher risk International Prognostic Scoring System (IPSS) score. The DFS was 65.5% at 2 years, and the CI of relapse was 27.6%. Meanwhile, the 2-year CI of grade I–III aGVHD was 31.5% (36). In another retrospective study analyzing 77 high-risk AML/MDS patients based on unfavorable genomic or clinical status at transplantation (AML, *n* = 54; MDS, *n* = 23), prophylactic or pre-emptive azacitidine combined with DLI achieved a 2-year CI of relapse of 22%, and the CIs of grade II–IV acute GVHD and cGVHD were 27.4 and 45%, respectively (77).

Like azacitidine, the use of decitabine in combination with DLI has been for both post-transplant salvage and maintenance in AML/MDS. A retrospective multicenter analysis from Germany reported that decitabine plus DLI achieved an ORR of 25% and CR of 17% in 36 patients with relapsed AML (*n* = 29) or MDS (*n* = 7). Meanwhile, only 7 patients (19%) experienced aGVHD after treatment. It is however noteworthy that the 2-year OS was only 11% in this cohort (96). In another retrospective analysis, including 26 patients with relapsed hematological malignancies (AML, *n* = 18, MDS, *n* = 6, MPN, *n* = 2), decitabine plus DLI achieved an ORR of 19% and a CR/CRi rate of 15% (97). In a prospective single-arm study, decitabine plus DLI was applied as prophylaxis in 28 patients with high-risk hematological malignancies, defined as with at least one of the unfavorable gene mutations (FLT3- ITD, TP53, ASXL1, DNMT3A, or TET2) (AML, *n* = 23; MDS, *n* = 2; ALL, *n* = 3). A 3-year relapse-free survival of 48.2% and OS of 48.9% were observed, together with a 3-year relapse rate of 26.1% post-DLI. Meanwhile, the incidence of grade II–IV aGVHD at 100 days post-DLI was 25.8%, and the cGVHD incidence at 3 years post-DLI was 21.6% (90). Collectively, the HMA/DLI combination after allo-HCT is safe for both consolidation and salvage therapy, while the efficacy remains to be further improved.

### FLT3 Inhibitors

FLT3 internal tandem duplications (ITDs) and FLT3-tyrosine kinase domain (TKD) mutations occur in ~25 and <10% of AML patients, respectively (105). FLT3 mutations, especially FLT3-ITD, indicate an adverse prognosis (106). Sorafenib is currently the only FLT3 inhibitor intensively investigated in the post-transplant setting. Single-agent sorafenib was shown to significantly reduce the relapse rate as maintenance therapy for FLT3-ITD mutant AML after allo-HCT (107, 108). Battipaglia et al. reported 27 patients with FLT3-positive AML who received sorafenib maintenance after allo-HCT, which led to a 1-year PFS of 92% (107). We observed that maintenance sorafenib was superior to prophylactic DLI in FLT3-ITD AML patients, with a lower relapse rate (4.2 vs. 25%) and a lower incidence of grade II–IV aGVHD (8.7 vs. 46.3%) (109).

The effects of post-transplant sorafenib is multifaceted, including direct FLT mutation inhibition, GVL effect augmentation through IL-15 production in FLT3-ITD mutant leukemia cells, and also synergic effects with alloreactive T cells (110). A synergic effect of sorafenib and DLI is therefore anticipated. Xuan et al. analyzed 83 FLT3-ITD mutant AML patients with overt relapse after allo-HCT who received salvage therapies (98). A superior survival was observed in sorafenib-containing regimen to conventional therapy (OS: 46.8 vs. 20%). In a subgroup analysis, the best survival was achieved for patients receiving sorafenib-containing chemotherapy followed by DLI, which is superior to other therapeutic regimens, including sorafenib combined with chemotherapy, chemotherapy followed by DLI, and monochemotherapy. No significant differences were observed concerning GVHD incidences among these therapies. In addition, Bruzzese et al. reported, in a small cohort of four patients, that the pre-emptive use of sorafenib followed by DLI in MRD-positive FLT3-ITD AML patients achieved MRD negativity in 3 patients (75%) (99). Interestingly, in this report, the remaining patient discontinued sorafenib because of toxicity and changed to gilteritinib, and long-term remission was achieved. Therefore, the use of other FLT3 inhibitors before and after allo-HCT as well as the combined use with DLI warrants further investigations.

### Bcl-2 Inhibitors

Aberrant Bcl-2 overexpression is identified in patients with AML, rendering survival advantage for the leukemia cells. The Bcl-2 inhibitor venetoclax combined with HMAs has achieved ~70% CR or CRi rate in untreated elderly AML patients unfit for conventional intensive chemotherapy (111, 112). In the post-transplant setting, Schuler et al. retrospectively analyzed 32 AML patients who relapsed after allo-HCT and reported an ORR of 47% after venetoclax/HMA combined therapy. However, 72% of patients experienced severe infections, and 78% of patients died after a median follow-up of 8.4 months (113). We have recently reported 44 patients (AML, *n* = 34) with post-allo-HCT relapse who received salvage venetoclax/HMA, which achieved a CR/CRi incidence of 34.1% (114).

The reports have shown that venetoclax enhances T cell-mediated cytotoxicity against AML (115) and does not impair activated T cell proliferation (116). Therefore, venetoclax plus DLI has been tried in the post-transplant setting. Amit et al. summarized 22 AML patients with post-transplant relapse who received the venetoclax/DLI combination; a total of 11 patients (50%) responded, and CR/CRi was achieved in 5 patients (23%). Meanwhile, microbiology-documented infections occurred in 8 patients (36%) and aGVHD in 4 patients (18%) (92).

### Other Agents

The deacetylase inhibitors (DACi) may enhance leukemia-specific cytotoxicity and mitigate GVHD but, conversely, could impair T and NK cell function (100). DACi panobinostat was evaluated in a phase I/II trial as maintenance therapy for high-risk AML/MDS in hematological complete remission after allo-HCT. High-risk AML was defined as with adverse risk cytogenetics, R/R disease, or secondary AML. In this study, 18 patients received the panobinostat/DLI combination, which showed a good safety profile but whose efficacy was unevaluable (100). Another phase I/II study evaluated the panobinostat/decitabine combination as maintenance in 110 patients with poor-risk AML/MDS after allo-HCT, in which 60 patients received DLI afterwards. The CI of relapse for the whole cohort was 35%, and the 2-year progression-free survival was 49%. Grades 3 and 4 adverse events related to panobinostat and decitabine were observed in 26% of evaluated patients. This study revealed the feasibility of the DACi/HMA/DLI combination as maintenance for AML after allo-HCT with enough safety and efficacy (101). Recently, IDH1/IDH2 inhibitors have been the other hotspot in the field of AML treatment. Instead of toxicity, IDH1/IDH2 inhibitors mainly exert therapeutic effects through the differentiation and maturation of malignant cells (117). In two pilot phase I studies, IDH1 inhibitor ivosidenib (118) achieved an ORR of 41.6%, and IDH2 inhibitor enasidenib (119) obtained an ORR of 40.3% in R/R AML patients with relevant mutations. Of note is that the continuous daily use of these two drugs led to a low frequency of treatment-related adverse events. The combined use of IDH1/IDH2 inhibitors and DLI requires further investigation for post-transplant AML relapse harboring these specific mutations.

## DLI Composition Manipulation and Engineering

### Donor-Derived NK Cells

The clinical studies on DLI composition manipulation and engineering are summarized in **Table 2**. Donor-derived natural killer (NK) cells may eliminate recipient malignant cells in the setting of mismatched or haploidentical transplant through alloreactivity (133) and attenuate GVHD *via* recipient dendritic cell elimination (134) and direct lysis or regulation of alloreactive T cells (135, 136). In a phase I trial, mbIL21 *ex vivo*-expanded donor NK cells were infused on days -2, +7, and +28 posttransplant for 13 patients with high-risk myeloid malignancies (AML, *n* = 8) who received PT-Cy-based haplo-HCT (120). In this study, high-risk AML was defined as a refractory disease or with unfavorable cytogenetics/molecular mutations, and high-risk MDS was that with an intermediate- or high-risk IPSS score. The safety of donor NK cell infusion was demonstrated, and it was observed that donor NK cell infusion might be related to lower viral infections and relapse rate. The other phase I trial tested prophylactic IL-2 activated donor-derived NK cell infusions 60–120 days after matched sibling allo-HCT in 16 patients with hematological diseases (AML, *n* = 6). The safety of this strategy was demonstrated, and promising efficacy was indicated (137). Meanwhile, Jaiswal et al. observed that a well-designed CD56-enriched DLI infusion after PT-Cy-based haplo-HCT prompted the good reconstitution of mature NK cells with a reduced incidence of aGVHD (121). More recently, CTLA4Ig has emerged as a novel and simpler approach in dissociating GVL and GVHD effects due to the rational that CTLA4Ig attenuates T cell activation but is resistant to NK cells. CTLA4Ig has been applied in combination with early sequential mDLI in patients with advanced hematological malignancies who received PT-Cy-based haplo-HCT. Compared to DLI alone, the CTLA4Ig-DLI decreased the acute GVHD (aGVHD) incidence from 18.8 to 9.6% and the cGVHD incidence from 36.5 to 15.3% (122). Interestingly, the disease progression rate was also lower in the CTLA4Ig-DLI group than in the DLI group (15.7 vs. 31.1%), which is probably due to an early expansion of functionally competent adaptive NK cells after the CTLA4Ig treatment (138, 139). Several strategies have been applied to enhance the anti-leukemic capacity of donor-derived NK cells, including the combined use with an immunomodulatory drug (eg., lenalidomide) (140), cytokine pre-activation (141), blocking of the inhibitory receptors [eg., PD1/PD-L1 (142) and iKIR (143)], selection of HLA-mismatched single KIR NK cells (144), or genetically modified NK cells (e.g., CAR-NK cells will be introduced in the next chapter). These techniques are expected to be used soon to potentiate NK cell therapy for AML relapse after allo-HCT.

**Table 2:** Studies on DLI composition manipulation/engineering for AML relapse after allo-HCT.

Study	No. of Patients (PerSpective/Retrospective)allo-HCT regimen	Diagnosis/Disease Status Before Therapy	Cellular Product/Drug	Technique Used	Cell or drug Course/Dose for Each Course	Toxicity	Disease Response	GVHD	Survival	Notes
**Donor-derived NK cells**										
Ciurea et al. (120)	13 (perspective) PT-Cy-based haplo-HCT	AML, *n* = 8 CML, *n* = 5 Remission	*Ex vivo* expanded donor NK cells	Expansion with mbIL21-expressing feeder cells	Median course number: 3 1 × 10^5^/kg to 1 × 10^8^/kg NK cells per course	No infusion reaction or DLTs	Relapse: 7.7%	Grades I–II aGVHD = 54% cGVHD = 0%	Follow-up: 14.7 months OS: 84.6% PFS: 84.6%	Infusions on days -2, +7, and +28 post-transplant
Jaiswal et al. (121)	10 (perspective) PT-Cy-based haplo-HCT	AML, *n* = 7 IMF, *n* = 1 CML, *n* = 1 MPAL, *n* = 1 Remission	CD56-enriched DLI	*Ex vivo* positive selection of CD56^+^ cells	Single infusion Median CD56^+^CD3^-^ cell count per dose: 6.7 × 10^6^/kg	No Infusion-related toxicities; DLT: grade 2 mucositis in 3 patients	1-year CI of relapse: 52%	aGVHD = 0% cGVHD = 32.2%	1-year OS: 50%	Single infusion 72 h after PT-Cy
Jaiswal et al. (122)	75 (prospective) PT-Cy-based haplo-HCT	AML/CML-BC, *n* = 32 ALL, *n* = 29 Lymphoma, *n* = 14 Remission	CTLA4Ig	N.A.	3 infusions on day +7, +21, and +35 Day +7: 1 × 10^6^ T cells/kg Day +21: 5 × 10^6^ T cells/kg Day +35: 5 × 10^6^ T cells/kg	N.A.	CTLA4Ig+DLI 2-year CI of relapse: 15.7%	CTLA4Ig+DLI 100-day CI of grade II–IV aGVHD: 9.6% 2-year CI of cGVHD: 15.3%	CTLA4Ig+DLI 2-yr OS: 83% 2-ye GRFS: 76.6%	mDLI infused 12 h after each CTLA4Ig infusion
**CAR-T cells**										
Cui et al. (123)	6 (prospective) ATG-based allo-HCT	AML, *n* = 6 Hematological relapse	CD38-directed CAR-T	N.A	Median course number: 1 Median cell dose per patient: 8.05 × 10^6^	CRS: 100% Grade 3 CRS: 16.7%	4 weeks after CAR-T: CR: 66.7% CRi: 66.7%	aGVHD and cGVHD: 0%	Median OS: 7.9 months; median LFS: 6.4 months	Fludarabine and cyclophosphamide regimen prior to CAR-T
**Other techniques**										
Roy et al. (124)	23 (prospective) TCD haplo-HCT	AML, *n* = 16 ALL, *n* = 7 Remission	ATIR101	*Ex vivo* alloreactive T cell depletion using photodepletion	Single infusion Cell dose: 2 × 10^6^ T cells/kg	N.A.	N.A.	2–4 aGVHD 17.4 4.3	1-year OS: 60.9%; 1-year GRFS: 56.5%	Donor unstimulated PBMC-derived ATIR101 Infusion: +28 day
Davies et al. (125)	19 (prospective) TCD haplo-HCT	AML, *n* = 12 ALL, *n* = 4 MDS, *n* = 3 Remission	Alloanergized DLI	Co-culture of unstimulated donor PBMC with gamma-irradiated allostimulator PBMC	Single infusion with dose escalation (*n* = 16) 10^3^ T cells/kg (*n* = 4) 10^4^ T cells/kg (*n* = 8) Dose level 3 10^5^ T cells/kg (*n* = 4)	DLTs: VOD, *n* = 1 respiratory distress syndrome, *n* = 5 sepsis/multi-organ failure, *n* = 1	Relapse incidence: 12.5%	aGVHD: 26.3% (5/19) cGVHD: 21.1% (4/19)	1-year OS: 38%	Alloanergized DLI on day +35 after transplant
Maung et al. (126)	16 (prospective) Alemtuzumab- or ATG-based HLA-identical allo-HCT	AML, *n* = 6 NHL, *n* = 4 MM, *n* = 2 HD, *n* = 1 CLL, *n* = 1 IMF, *n* = 1 MDS/MPN, *n* = 1 Remission	CD45RA^+^ naive T cell-depleted DLI	*Ex vivo* depletion using magnetic particles	Single infusion with dose escalation for each patient 1 × 10^6^ T cells/kg 5 × 10^6^ T cells/kg 1 × 10^7^ T cells/kg	No DLTs	Relapse incidence: 43.7%	CI of aGVHD: 6.2% CI of cGVHD: 6.2%	2-yr OS: 68.8%; 2-yr PFS: 50%	Infusion at least 60 days after transplant
Ho et al. (127)	16 (prospective) allo-HCT from MSD donors	HL, *n* = 5 CLL, *n* = 3 MPN/MF, *n* = 3 NHL, *n* = 2 AML, *n* = 1 MDS, *n* = 1 MM, *n* = 1 Hematological relapse	Co-infusing donor-derived DC and DLI	DC generated *ex vivo* from donor PBMCs	Single DC+DLI infusion Median DC yield: 1.16 × 10^8^ cells DLI: 3 × 10^7^ T cells/kg	DLTs: 1 idiopathic respiratory failure; 1 ventricular cardiac arrest	ORR: 28.6% (4/14) CR: 21.4% (3/14)	aGVHD: 7.1% cGVHD: 7.1%	8-year OS: 43.8%	DLI infused 24 h after DC infusion
Laport et al. (128)	18 (prospective) allo-HCT from MSD donors (protocol unknown)	NHL, *n* = 5 MM, *n* = 3 AML, *n* = 2 ALL, *n* = 2 CLL, *n* = 2 MDS, *n* = 2 APL, *n* = 1 HD, *n* = 1 Hematological relapse	CIKs	Major cytokines added: CD3 mAb and IL-2	Single infusion with dose escalation for each patient 1 × 10^7^ T cells/kg (*n* = 4) 5 × 10^7^ T cells/kg (*n* = 6) 1 × 10^8^ T cells/kg (*n* = 8)	DLTs: 1 sustained ventricular tachycardia 1 hepatic transaminase level rises	CR: 27.8% (5/18)	aGVHD: 11.1% (2/18) cGVHD: 5.6% (1/18)	Median OS: 28 months Median EFS: 4 months	
Narayan et al. (129)	44 (prospective) TLI-ATG-based allo-HCT	AML, *n* = 12 MDS, *n* = 27 MPN, *n* = 2 MDS/MPN, *n* = 3 Remission	CIKs	Major cytokines added: CD3 mAb and IL-2	Single infusion 1 × 10^8^ T cells/kg	N.A.	2-year CI of relapse: 65.9%	100-day CI of aGVHD: 20.5% 2-year CI of cGVHD: 28.2%	2-year OS: 50.6% 2-year EFS: 27.3%	CIKs infused between day +21 and day +35 after allo-HCT
Merker et al. (130)	36 (prospective) Alemtuzumab or ATG based allo-HCT	AML, *n* = 15 ALL, *n* = 18 NHL, *n* = 2 CML, *n* = 1 Hematological/molecular relapse	CIKs	Major cytokines added: IL-15, IFN-γ, anti-CD3 mAb, IL-2	Each patient received: dose escalation initiated from 1 × 10^6^/kg to 5 × 10^6^/kg, 1 × 10^7^/kg to a maximum of 1 × 10^8^ T cells/kg	N.A.	CR: 53%	aGVHD: 25% cGVHD: 6%	6-month OS: 77%	
Introna et al. (131)	73 (prospective) Allo-HCT with different regimens	AML, *n* = 41 ALL, *n* = 19 MM, *n* = 4 HD, *n* = 3 NHL, *n* = 2 MDS, *n* = 2 MPN, *n* = 2 Hematological/molecular relapse	Sequential infusion of DLI and CIKs	Major cytokines added: CD3 mAb and IL-2	Each patient received: 2 DLI infusions plus 3 CIK infusions with dose escalation Each DLI 1 × 10^6^/kg CIK initial dose: 1 × 10^6^/kg OR 5 × 10^6^/kg	No DLTs	ORR: 30% CR: 26%	100-day CI of aGVHD: 12% 3-year CI of cGVHD: 17%	1-year OS: 51% 1-year PFS: 31%	CIK starts 3 weeks after second DLI
Williams et al. (132)	10 (prospective) Transplant protocol unknown	AML, *n* = 5 B-ALL, *n* = 3 HD, *n* = 2 Hematological relapse	TAA-T	Donor T lymphocytes expanded with donor APCs with TAA pepmixes	Each patient received: 1–3 doses of TAA-T at sequential dose levels up to 4.0 × 10^7^/m^2^ per dose	N.A.	ORR: 78% CR: 44%	No GVHD	N.A.	

Allo-HCT, allogeneic hematopoietic cell transplantation; GVHD, graft-versus-host disease; PT-Cy, post-transplantation cyclophosphamide; haplo-HCT, haploidentical hematopoietic cell transplantation; AML, acute myeloid leukemia; CML, chronic myeloid leukemia; DLTs, dose-limiting toxicities; aGVHD, acute GVHD; cGVHD, chronic GVHD; OS, overall survival; PFS, progression-free survival; IMF, idiopathic myelofibrosis; MPAL, mixed-phenotype acute leukemia; DLI, donor lymphocyte infusion; CI, cumulative incidence; CML-BC, CML blast crisis; CAR-T, chimeric antigen receptor T cell; CRS, cytokine releasing syndrome; CR, complete remission; CRi, CR with incomplete count recovery; LFS, leukemia-free survival; GRFS, GVHD-free, relapse-free survival; MDS, myelodysplastic syndrome; VOD, veno-occlusive disease; MM, multiple myeloma; HD, Hodgkin’s disease; CLL, chronic lymphocytic leukemia; MPN, myeloproliferative neoplasms; TAA-T, tumor-antigen-specific T cells.

### CAR-T and CAR-NK Cells

CAR-T infusions post-allo-HCT have been intensively investigated, especially in B-ALL. Both allogenic and autologous CAR-T cells targeting CD19 (CART19) have been applied in clinical trials for B-ALL relapse after allo-HCT, with a CR rate of more than 80% (145) (146, 147). Apart from the satisfactory remission rate, CAR-T seems to confer a lower aGVHD incidence compared to DLI. Smith et al. summarized 132 patients from 9 studies who received posttransplant CART19 and observed a total aGVHD rate of 14% for donor-derived CAR-T and only 2% for recipient-derived CAR-T (148).

CAR-T cell therapy in AML has been much more challenging compared to B cell malignancies, majorly due to the lack of AML-specific target antigens and clonal heterogeneity, leading to unwanted on-target off-leukemia toxicity and risk of relapse from minor clones (149). Despite the difficulties, major efforts have been made to develop CAR-T cells for AML. Cui et al. reported a pioneering study using CD38-directed CAR-T cells in 6 patients with relapsed CD38^+^ AML after allo-HCT, and 4 (66.7%) achieved CR or CR with incomplete count recovery (CRi) (123). Notably, several key CAR-T products have shown promising efficacy in R/R AML and are tested in clinical trials [summarized by Daver et al. (150)]. An earlier study showed that LeY CAR-T achieved 1 CR in 4 adult R/R AML patients (151). The CD33 antibody–drug conjugate gemtuzumab ozogamicin was the only approved antibody-targeted therapy for AML, and the CD33/CD3-bispecific BiTE antibody construct has shown potent pre-clinical anti-AML activity (152). Although an earlier case report showed unsatisfactory results in one refractory AML patient receiving CD33 CAR-T infusion (153), several clinical trials are ongoing to prove its efficacy. CD123 CAR-T has shown potent antileukemic efficacy in a preclinical human AML xenograft model, while severe hematologic toxicities accompany the conventional second-generation CD123 CAR-T (154). More recently, a switchable universal CAR-T platform (UniCAR) has been tested in clinical trials (155). In a preliminary report on 3 R/R AML patients receiving CD123 UniCAR-T, the cell infusion was well tolerated, and CRi was achieved in 2 patients and PR in 1 patient (156). In addition, NKG2D CAR-T cells have been evaluated in a phase I clinical trial on AML/MDS and multiple myeloma patients. Among the 7 reported R/R AML patients, the CR/CRi rate was 42% (157). Moreover, CAR-T cells redirected to several novel targets [FLT3 (158, 159), CLL-1 (160), CD70 (161), IL-10R (162), mesothelin (163), Siglec-6 (164), *etc.*] have shown potent efficacy in pre-clinical studies, and some of them (eg., CLL-1) are currently being assessed in clinical trials (150). Furthermore, to overcome AML heterogeneity, dual CAR-T cells have also been investigated in AML. In a phase I study, Liu et al. evaluated compound CAR-T cells targeting both CD33 and CLL-1 in R/R AML; 2 patients who had blast counts >20% before cCAR T cell infusion achieved MRD-negative remission and were able to proceed to allo-HSCT (165). Other dual CAR-T, such as CD123/CLL-1 CAR-T cells, and further engineered CAR-T cells, such as CD19 CAR-T cells engineered to secrete a biparatopic anti-CLEC12A bridging protein, have been developed and evaluated (166).

Despite its potency, CAR-T cell therapy owns well-defined limitations, including cytokine release syndrome (CRS) and GVHD. With the potential to dissociate the GVL and GVHD effects, CAR-NK cells potentially confer better disease control with lower toxicity than CAR-T cells in both pre- and post-transplant settings (167). Due to difficulties in manufacturing (168), CAR-NK has been forwarded to clinical trials [summarized by Lu et al. (169)] more lately than CAR-T cells. Following the success of CD19 CAR-NK cells on B cell malignancies (170), few but encouraging results have been published on AML. Tang et al. reported three patients with R/R AML who received CD33-CAR NK-92 cells in a phase I trial. One patient experienced a high fever and two moderate fevers, and grade I CRS was observed in 2 patients, showing enough safety for the CAR-NK cell infusions in patients with high tumor burden (171). Moreover, CAR-NK redirected to several other AML antigens [*e*.*g*., NKG2D ligands (172), CD38 (173), CD123 (174), NPM1c (175), CD7 (176), etc.] have shown preclinical efficacy in hematological malignancies and are being investigated in clinical trials for AML.

## Other Techniques of DLI Composition Manipulation and Engineering

Efforts have been made to modify the composition of DLI to reduce the GVHD rate but maintain the GVL effects. Infusion of ATIR101, the donor-derived T cell-enriched product selectively depleted of recipient-alloreactive T cells, in T cell-depleted (TCD) haplo-HCT has decreased the NRM rate and improved the GRFS versus TCD haplo-HCT alone (124). In addition, infusion of alloanergized DLI generated *ex vivo* on day +35 was shown to promote immune reconstitution and expand regulatory T cells after CD34-selected haplo-HCT (125). Inspired by the preclinical studies which indicated that naïve T cells are major drivers of GVHD, a phase I dose escalating study of CD45RA^+^ naïve T cell-depleted DLI was conducted in allo-HCT patients. Among the 28 patients evaluated, no patient developed grade III/IV acute GVHD or severe cGVHD, indicating the preventive capacity of this technique against GVHD (126). Moreover, since dendritic cells may bolster T cell responses through antigen presentation, a phase I trial has evaluated the co-infusion of DC followed by DLI in 16 relapsed patients with hematological malignancies after allo-HCT. Among the 14 evaluable patients, 4 (29%) achieved long-term remission, and only one developed grade II aGVHD (127).

Cytokine-induced killer cells (CIK cells), which are *ex vivo*-activated cytotoxic T cells containing predominantly CD3^+^CD8^+^NKG2D^+^ cells along with significantly expanded CD3^+^CD56^+^ cells, have shown anti-leukemic activity without causing severe aGVHD in relapse treatment (128) or consolidation (129) after allo-HCT. Merker et al. observed superior disease control but lower incidence of aGVHD for CIK in a comparative study between CIK and DLI in patients with overt hematologic relapse after allo-HCT (130). The sequential infusion of DLI and CIK has also been tested in a phase II trial for hematological relapse post-allo-HCT, but the response rate was unsatisfactory (response rate, 30%) (131). Notably, CAR-engineered CIK cells have shown potent anti-leukemic efficacy in pre-clinical studies *in vitro* and *in vivo* (177). Another attractive modality is multiple tumor antigen-specific T cells (TAA-T). In a prospective study conducted by Williams et al., expanded lymphocytes reactive to TAAs, including WT1, PRAME, and surviving, achieved 60% (3/5) CR rate in 5 AML patients relapsed after allo-HCT without causing GVHD (132). Moreover, T cells engineered with specific TCR (TCR-T) have shown potent anti-leukemic efficacy *in vitro* and in xenograft mouse models of lymphoid malignancies with low risk of aGVHD (178, 179). Finally, adoptive transfer of suicide gene-modified DLI has allowed efficient GVHD control *via* inducible cell elimination (180, 181).

## Conclusions and Perspectives

The aim of DLI optimization is to maximize its GVL while minimizing the GVH effects. Many factors, including transplantation type, donor origin, disease burden, DLI dosage/timing, and immunosuppression, all affect greatly its efficacy/toxicity. It is recommended to use prophylactic/pre-emptive DLIs especially in high-risk AML after allo-HCT since therapeutic DLI for overt relapse is related to a lower chance of disease control. MRD-guided pre-emptive DLI awaits further improvement with the introduction of unbiased techniques with higher specificity, such as whole-genome sequencing. Additional use of novel drugs and composition manipulation are two promising directions which may revolutionize the DLI. Many novel targeted/immunomodulatory drugs are in the pipeline for clinical use in AML, while their efficacy/toxicity as well as their influence on the GVL/GVH effects must be clarified before clinical use after allo-HCT. Finally, cell engineering may help to realize the final aim of “perfect DLI” with full disease control and no GVHD, which awaits breakthrough in cellular immunotherapy for myeloid diseases. Integration of novel techniques (eg., CAR and TCR combined use) and personalized cell engineering techniques may further enhance the efficacy of cellular immunotherapy.

## Author Contributions

YL and YY designed this study. YY and LY wrote the manuscript. YL and HH revised each section and gave expert comments. All authors contributed to the article and approved the submitted version.

## Funding

This work was supported by the National Science Foundation of China (grant # 82000180 and grant # 82170205).

## Conflict of Interest

The authors declare that the research was conducted in the absence of any commercial or financial relationships that could be construed as a potential conflict of interest.

## Publisher’s Note

All claims expressed in this article are solely those of the authors and do not necessarily represent those of their affiliated organizations, or those of the publisher, the editors and the reviewers. Any product that may be evaluated in this article, or claim that may be made by its manufacturer, is not guaranteed or endorsed by the publisher.
